# Comparison of a web-based food record tool and a food-frequency questionnaire and objective validation using the doubly labelled water technique in a Swedish middle-aged population

**DOI:** 10.1017/jns.2016.29

**Published:** 2016-10-03

**Authors:** Sanna Nybacka, Heléne Bertéus Forslund, Elisabet Wirfält, Ingrid Larsson, Ulrika Ericson, Eva Warensjö Lemming, Göran Bergström, Bo Hedblad, Anna Winkvist, Anna Karin Lindroos

**Affiliations:** 1 Department of Internal Medicine and Clinical Nutrition, Institute of Medicine, Sahlgrenska Academy, University of Gothenburg, Gothenburg, Sweden; 2 Department of Clinical Sciences in Malmö, Research Group in Nutritional Epidemiology, Lund University, Lund, Sweden; 3 Department of Endocrinology, Diabetology and Metabolism, Sahlgrenska University Hospital, Gothenburg, Sweden; 4 Department of Clinical Sciences in Malmö, Diabetes and Cardiovascular Disease, Genetic Epidemiology, Lund University, Lund, Sweden; 5 National Food Agency, Uppsala, Sweden; 6 Wallenberg Laboratory, Sahlgrenska Centre for Cardiovascular and Metabolic Research, Sahlgrenska University Hospital, Gothenburg, Sweden; 7 Department of Clinical Sciences in Malmö, Cardiovascular Research Group, Lund University, Lund, Sweden

**Keywords:** Diet assessment, Food records, FFQ, Doubly labelled water, Validation, Web-based methods, Nutrition epidemiology, DLW, doubly labelled water, EI, energy intake, SCAPIS, Swedish CArdioPulmonary bioImage Study, TEE, total energy expenditure, TEE_DLW_, total energy expenditure measured with the doubly labelled water technique

## Abstract

Two web-based dietary assessment tools have been developed for use in large-scale studies: the Riksmaten method (4-d food record) and MiniMeal-Q (food-frequency method). The aim of the present study was to examine the ability of these methods to capture energy intake against objectively measured total energy expenditure (TEE) with the doubly labelled water technique (TEE_DLW_), and to compare reported energy and macronutrient intake. This study was conducted within the pilot study of the Swedish CArdioPulmonary bioImage Study (SCAPIS), which included 1111 randomly selected men and women aged 50–64 years from the Gothenburg general population. Of these, 200 were enrolled in the SCAPIS diet substudy. TEE_DLW_ was measured in a subsample (*n* 40). Compared with TEE_DLW_, both methods underestimated energy intake: −2·5 (sd  2·9) MJ with the Riksmaten method; −2·3 (sd 3·6) MJ with MiniMeal-Q. Mean reporting accuracy was 80 and 82 %, respectively. The correlation between reported energy intake and TEE_DLW_ was *r* 0·4 for the Riksmaten method (*P* < 0·05) and *r* 0·28 (non-significant) for MiniMeal-Q. Women reported similar average intake of energy and macronutrients in both methods whereas men reported higher intakes with the Riksmaten method. Energy-adjusted correlations ranged from 0·14 (polyunsaturated fat) to 0·77 (alcohol). Bland–Altman plots showed acceptable agreement for energy and energy-adjusted protein and carbohydrate intake, whereas the agreement for fat intake was poorer. According to energy intake data, both methods displayed similar precision on energy intake reporting. However, MiniMeal-Q was less successful in ranking individuals than the Riksmaten method. The development of methods to achieve limited under-reporting is a major challenge for future research.

In nutrition research, a major limitation is the notorious difficulty in measuring dietary intake with techniques that are precise, accurate and applicable to large numbers of free-living individuals^(^^1^^,^^2^^)^. All dietary assessment methods have strengths and limitations which need to be considered when choosing a dietary assessment method and analysing data in a study. Since erroneous food intake data could lead to false interpretations when evaluating diet–disease associations or calculating toxicological hazards, it is of major importance that the dietary assessment tools used in large population studies are up to date and validated for what they are intended to measure^(^^3^^)^.

Food records have long been considered the most accurate and detailed method for measuring dietary intake^(^^4^^,^^5^^)^. A major advantage with food records is the open format, which makes it possible to capture detailed information on food choices and timing of eating during the registration period. However, a few recording days may not be representative of habitual food intake or seasonal variations unless repeated several times over the year^(^^6^^,^^7^^)^. In addition, food records are time consuming for both participants and staff and are therefore expensive to administer in large-scale studies, and there is also the possibility that participants might deviate from their normal eating habits during the recording days. In contrast, FFQ are relatively easy to apply and the time burden is reduced for both participants and study staff. FFQ are therefore commonly used in large-scale epidemiological studies despite being less accurate and detailed. In recent years, the integration of repeated 24-h recalls together with FFQ has been discussed as a solution to improve the level of dietary data obtained^(^^8^^)^.

Advances in technology have enabled the development of innovative ways of measuring food intake and new methods have been developed which allow participants to report their dietary intake via the web^(^^9^^–^^12^^)^. There are many advantages with collecting data via the web compared with using traditional paper-based methods. It is easy for the study participants to access the questionnaire at any time and location, and time is saved when the participants enter their information themselves. In addition, there is no need of coding the data. The rapid improvements in advanced technology could potentially improve the quality of collected dietary data, and one major challenge lies in the development of methods that manage to minimise under-reporting of dietary intake. However, so far only a few web-based dietary assessment tools have been validated against objectively measured energy expenditure using the ‘gold standard’, doubly labelled water (DLW) technique.

Recently, two web-based dietary assessment methods have been developed for use in large-scale studies: the Riksmaten method, which is a web-based 4-d food record, and the more rapid food-frequency method MiniMeal-Q^(^^13^^,^^14^^)^. The Riksmaten method was developed for the national dietary surveys in Sweden and has so far only been validated on diet quality parameters^(^^15^^,^^16^^)^. MiniMeal-Q was developed for use in large-scale epidemiological studies and has previously been validated on energy, and macro- and micronutrients using truncated data from the longer-version Meal-Q. The validation process was performed in a relatively young (mean age 33 years), highly educated and predominantly female group and the objective validation with DLW showed a correlation on *r* 0·38 on energy intake (EI)^(^^13^^)^. Both methods are currently widely used in epidemiological research and there is a need to validate these two methods in a middle-aged population with participants living in areas with different socio-economic status (SES). Thus, the aim of this study was to validate these two methods by reported EI against objectively measured total energy expenditure (TEE) using the DLW technique. A relative comparison of the two methods on reported energy and macronutrient intake was also performed.

## Materials and methods

### Study population

**Fig. 1.**
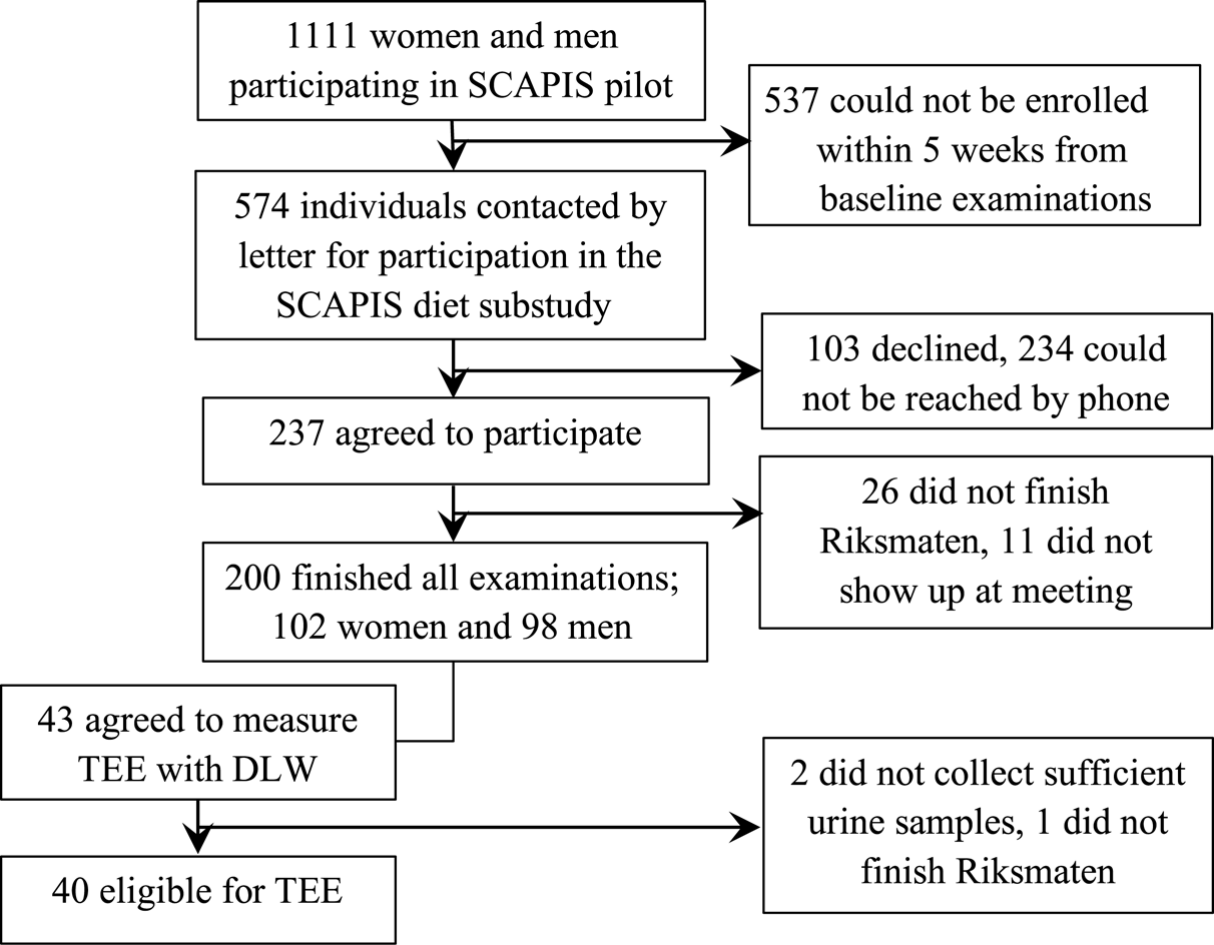
Flow chart of recruitment of participants to the Swedish CArdioPulmonary bioImage Study (SCAPIS) diet substudy. TEE, total energy expenditure; DLW, doubly labelled water.

This study takes advantage of the extensive collection of clinical data, blood samples and dietary data assembled during 2012 within the pilot study of SCAPIS (Swedish CArdioPulmonary bioImage Study)^(^^17^^)^. Men and women aged 50–64 years from the Gothenburg general population were invited to participate in the SCAPIS pilot study. A computerised random selection was made from two different areas in Gothenburg, which were classified as either low or high SES, respectively. A total of 2243 men and women were invited and 1111 men and women gave informed consent to participate in the pilot study, which included comprehensive clinical measurements, anthropometry, bioimaging and blood sampling during the time period from February 2012 to December 2012. Participants who had finished all clinical examinations, and who were expected to be recruited within the time frame of 5 weeks from the baseline examinations of the SCAPIS pilot study, received a letter inviting them to take part in the SCAPIS diet substudy at the Department of Internal Medicine and Clinical Nutrition, Sahlgrenska Academy. No exclusion criteria were applied for the initial recruitment, but it was a prerequisite to understand written Swedish for the diet substudy. The SCAPIS diet substudy aimed to validate two dietary assessment methods used in the SCAPIS study, by recruiting 100 women and 100 men consecutively during April to December 2012. In total, 237 (42 %) out of 574 invited subjects agreed to participate in the SCAPIS diet substudy, of whom forty-three also agreed to measure TEE with DLW. Of all requested participants, more women than men (46 *v*. 36 %), as well as participants living in high-SES compared with low-SES areas (49 *v*. 32 %), agreed to participate. In the final analyses 200 participants were eligible, of whom forty subjects (twenty women and twenty men, 53 % of high SES) also measured TEE with DLW (Fig. 1).

### Study design

Participants enrolled in the diet substudy were invited to a 1 h group meeting with a dietitian. At the visit participants first completed MiniMeal-Q, and were thereafter given instructions on how to complete the Riksmaten method at home during the oncoming week. Participants who had agreed to take part in the DLW substudy were invited to a revisit 14 d later. The TEE was thus measured during the same time period as the food recording, while the FFQ reflected the period of the past few months before the measurement. Participants were told to maintain a ‘normal’ activity level and diet during the study period. All data regarding anthropometry and background characteristics (Table 1) were collected at the SCAPIS pilot study examinations, whereas the main focus of the diet substudy was to collect dietary data. This study was conducted according to the guidelines laid down in the Declaration of Helsinki and all procedures involving human subjects were approved by the Gothenburg Regional Ethics Committee. Written informed consent was obtained from all subjects.

**Table 1.** Characteristics of the study population in the Swedish CArdioPulmonary bioImage Study (SCAPIS) diet substudy* (Mean values and standard deviations; numbers and percentages)

	Women (*n* 102)		Women DLW (*n* 20)			Men (*n* 98)		Men DLW (*n* 20)		
	*n*	%	*n*	%	*P*	*n*	%	*n*	%	*P*
Age (years)					0·970					0·335
Mean	57·7		57·8			57·7		58·6		
sd	4·5		4·1			4·7		4·9		
Weight (kg)					0·186					0·454
Mean	72·1		68·4			88·3		86·7		
sd	13·7		8·8			10·5		10·3		
BMI (kg/m^2^)					0·485					0·921
Mean	26·4		25·7			27·4		27·3		
sd	4·8		3·1			2·9		3·0		
BMI					0·346					0·997
<24·9 kg/m^2^	50	49	10	50		20	20	4	20	
25–29·9 kg/m^2^	37	36	9	45		63	64	13	65	
≥30+ kg/m^2^	15	15	1	5		15	15	3	15	
Country of birth					0·104					0·686
Sweden	81	79·4	14	70		75	76·5	15	75	
Nordic countries	11	10·8	5	25		6	6·1	2	10	
Non-Nordic origin	10	9·8	1	5		17	17·3	3	15	
Smoking: current smoker†	14	14	5	25	0·297	9	9	2	10	0·635
Education: university or college degree	43	42	6	30	0·501	37	38	8	40	0·363
SES area					0·698					0·168
Low	42	41	9	45		36	37	10	50	
High	60	59	11	55		62	63	10	50	

DLW, doubly labelled water; SES, socio-economic status.

* Data assembled within the SCAPIS pilot study.

† Missing data; smoking (*n* 1).

### Doubly labelled water

TEE was determined by the DLW technique. In weight-stable individuals TEE is equal to total EI, and therefore DLW provides an objective measure of TEE in free-living individuals^(^^18^^)^. The DLW procedure has been described in detail elsewhere^(^^19^^)^. In short, at the first visit body weight was measured and a spot urine sample was collected from each participant for determination of background isotope enrichment. Thereafter, an oral dose of DLW was administered of 1 g DLW/kg, corresponding to 0·05 g of ^2^H-labelled water (99·9 %) and 0·10 g of ^18^O-labelled water (10 %) per kg body weight. Because of the decreasing total body water content with increasing BMI, men with BMI >30 kg/m^2^ were given a dose of 0·6 g DLW/kg and women a dose of 0·5 g DLW/kg. The dose was flushed down the throat with a glass of tap water. A total of five 30 ml urine samples were collected; one prior to receiving the oral dose of DLW as reference, and four more at days 1, 3, 12 and 14. Participants were instructed to collect the second voiding of the day, to note down the exact time when the urine samples were collected and to seal the caps immediately after sampling. The last four samples were stored in the household freezer by each participant until the study end. Weight was also measured after the study period had ended to ensure weight maintenance. A weight difference of ±1 kg was considered to be acceptable as a reflection of normal biological variations in body weight. All analyses were made at Department of Internal Medicine and Clinical Nutrition in Gothenburg. Measurements of tap water, diluted administered dose, background enrichment and urine samples were performed in triplicate on a Finnigan MAT Delta Plus Isotope-Ratio Mass Spectrometer (ThermoFinnigan). Total daily energy expenditure was calculated by the multipoint method from the difference between elimination constants of hydrogen and oxygen, by linear regression, with the assumptions for fractionating as suggested by International Atomic Energy Agency^(^^20^^)^. All elimination curves were checked for major diverging residuals. The relationship between pool size ^2^H and pool size ^18^O was used as a quality measurement of the technique.

### The Riksmaten method

The Riksmaten method is a web-based 4 d food record, developed by the Swedish National Food Agency (NFA) prior to the Swedish national dietary survey in adults, Riksmaten 2010–2011^(^^21^^)^. The food list in the web tool (version 04.1) consisted of 1909 different food items and dishes, which are linked to the Swedish food composition database (Livsmedelsdatabasen, version Riksmaten adults 2010–2011) at the NFA, which enables automatic estimation of energy and nutrient intake. A simplified version of the Riksmaten method is to be found in Swedish at the NFA website^(^^22^^)^. To estimate portion sizes a portion guide was used which consisted of twenty-four different food categories, with four to eight different reference sizes in each category. It was available in the web tool as well as a printed portion guide. Participants received a personalised login to enter the web tool and were told to record all food and drinks consumed, and to estimate a portion size for each item according to the previously described portion guide. Dietary supplements taken during the recording days were reported in a separate form on the website. It was optional to enter the food intake via the web (which they were encouraged to do continuously), or to report the intake by telephone to the study dietitian. The printed portion guide, a paper diary and an information folder on how to keep food records were given to the participants. To ensure a more equal distribution among weekdays registered, participants were told to begin their registration either on the next coming Tuesday, Wednesday or Saturday. At 2 d after the diet record had begun, all participants were contacted by the dietitian by telephone to enhance compliance and provide an opportunity to ask questions. Based on the recorded intake of food and drinks, the average energy and nutrient intake per d was calculated for the current analyses. Participants also completed two questions about their physical activity level during the past 12 months at work and leisure time separately, on a four-grade scale. After completion all data were examined, and to be considered as an approved record at least two energy-containing meals a day should have been recorded. After submitting their registrations, participants received an overview of their energy and macronutrient intake as well as intake of selected micronutrients. Participants who reported their food intake by telephone received the same information by regular mail.

### MiniMeal-Q

The web-based FFQ MiniMeal-Q used in the study is a short form of Meal-Q^(^^13^^)^. The Meal-Q was developed at Institute of Medical Epidemiology and Biostatistics, Karolinska Institute, Sweden, and shortened to MiniMeal-Q for the LifeGene-project^(^^23^^)^. The questionnaire is self-administered, semi-quantitative and contains questions about meal patterns and portion sizes. It was developed to include follow-up questions only on food items that had been consumed at least once per month. The questionnaire consists of both single foods and mixed dishes and covers a time period of the past few months. Because of its dynamic structure, it includes between seventy-five to 126 food items. Most questions have an optional answering frequency in a nine-grade scale from ‘five times a day’ to ‘one-to-three times a month’. For the estimation of portion sizes on cooked dishes, five different photo-options are presented for: (1) meat, chicken, fish and vegetarian substitutes; (2) potatoes, rice and pasta; and (3) vegetables (both raw and cooked). Other foods are calculated by standard portion sizes. All dietary data were linked to the Swedish food composition database (Livsmedelsdatabasen, version 2012-01-06) and calculated as the average intake of unit/d.

### Statistical analysis

Characteristics for the whole study population and the subgroup that underwent DLW analyses, as well as comparisons between participants living in low- and high-SES areas, were compared using two-sided *t* test for equal means for continuous, normally distributed variables, independent-samples Mann–Whitney *U* test for non-normally distributed variables and χ^2^ test for categorical variables. EI from the Riksmaten method and MiniMeal-Q was pairwise compared with TEE measured with DLW (TEE_DLW_), and presented as the mean of the absolute difference (EI – TEE_DLW_) and percentage of reporting accuracy (EI:TEE_DLW_ × 100). Under-reporting of EI was based on the 95 % confidence limits of expected EI:TEE of 1^(^^24^^)^. The 95 % confidence limits were calculated as:

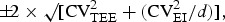

where CV_TEE_ is the within-subject CV for TEE (8·4 %) based on a time span of 2 weeks^(^^23^^)^, *d* is the number of days from food record (4 d) and CV_EI_ is the within-subject CV for EI from food record (20·6 %). This gives an interval where participants were considered as under-reporters of EI at EI:TEE <0·73, and over-reporters at EI:TEE >1·27 for the Riksmaten method. For FFQ the number of days is taken as infinite, whilst only CV_TEE_ is taken into account. The interval for MiniMeal-Q was therefore set at EI:TEE <0·83 for under-reporting and EI:TEE >1·17 for over-reporting. Pearson correlations between TEE_DLW_ and EI from both methods were calculated.

The mean and median intakes of total energy and nutrient intakes are presented for both sexes separately. The Wilcoxon signed-rank test was used to compare energy and nutrient intakes from both methods and Spearman correlation coefficients were calculated for the association between absolute energy and nutrient intake. Since the dietary variables were not normally distributed all variables were either log or square root transformed prior to further analyses. Pearson correlation coefficients were calculated on energy-adjusted nutrient intake variables, and the energy adjustment was performed by the residual model^(^^25^^)^. The absolute agreement between both methods was evaluated with Bland–Altman plots^(^^26^^)^. Here, both crude and energy-adjusted data were used, in this case expressed as the energy density (i.e. unit/MJ). The plot obtained illustrates the differences between the two measurements against the mean of both methods. A 95 % CI calculated as the mean difference ±1·96 sd allowed for the evaluation between the methods within the limits of agreement. The ability to rank individuals by energy and nutrient intakes (using energy-adjusted values) was examined by dividing the study population into tertiles for energy, and quartiles for nutrients. Through a cross-tabulation the Cohen's weighted κ (*κ*_w_) was obtained. All statistical analyses were two-sided with a significance level at *α* < 0·05. Statistical analyses were performed using SPSS Inc. (released 2009) PASW Statistics for Windows, version 18.0 (SPSS Inc. and IBM Corp.) and SAS 9.2 for Windows (SAS Institute, Inc.).

## Results

The mean age of both women and men was 58 years (range 50–65 years) and 15 % of both women and men had a BMI above 30 kg/m^2^. A majority of the women (79 %) and men (77 %) were of native Swedish origin and approximately 40 % of the participants were living in an area categorised as low SES (Table 1). No statistically significant differences could be found between the whole study population (*n* 200) and the subgroup participating in DLW analyses (*n* 40) on the background characteristics evaluated. Stratification on low-/high-SES areas showed that participants from low-SES areas had a significantly higher BMI (27·8 *v*. 26·3 kg/m^2^, *P* = 0·008), were more likely to be smokers (15 *v*. 5 %; *P* < 0·001), born outside Sweden (49 *v*. 5 %; *P* < 0·001) and had a lower education level (17 *v*. 56 % with university/college degree; *P* < 0·001) than participants living in areas categorised as high SES (data not shown). Most participants (93 %) reported their 4-d food records by the web system, and there were no differences in reported EI between web-records or telephone-records (*P* = 0·23). Most participants evaluated both methods as easy to understand and to use.

### Objective validation of energy intake

All participants who completed the DLW measurements (*n* 40) remained weight stable during the time frame of these measurements. The comparison of TEE_DLW_ in relation to reported EI from the Riksmaten method and MiniMeal-Q are displayed in Table 2. Combining women and men, the measured mean TEE_DLW_ was 10·8 (95 % CI 9·9, 11·6) MJ/d, which was significantly higher than reported EI from both the Riksmaten method and MiniMeal-Q (8·3 (95 % CI 7·5, 9·1) and 8·5 (95 % CI 7·4, 9·5)  MJ, respectively; *P* < 0·001). Linear regression of TEE_DLW_ *v*. reported EI showed a calibration coefficient of between 0·09 and 0·66 for the Riksmaten method, and between −0·04 and 0·72 for MiniMeal-Q. Mean reporting accuracy was 80 (sd 23) % for the Riksmaten method and 82 (sd 33) % for MiniMeal-Q. The correlation for measured TEE_DLW_ and reported energy was *r* 0·40 for the Riksmaten method (*P* < 0·05) and *r* 0·28 for MiniMeal-Q (ns). Of the participants, sixteen (40 %) were considered as under-reporters and one (5 %) as an over-reporter in EI with the Riksmaten method. For MiniMeal-Q, 23 participants (57·5 %) were considered as under-reporters and six (15 %) as over-reporters.

**Table 2.** Comparison of total energy expenditure measured with doubly labelled water (TEE_DLW_) in relation to reported energy intake (EI) from the Riksmaten method and MiniMeal-Q in a subgroup of twenty women and twenty men

	Energy (MJ)	95 % CI	EI:TEE_DLW_ correlation†	Calibration coefficient (*λ* value)‡	95 % CI	EI – TEE_DLW_ (MJ/d)	sd	EI:TEE_DLW_ (%)	sd
Riksmaten									
Women (*n* 20)	7·3	6·5, 8·1	0·33	0·29	−0·12, 0·70	−1·7**	2·1	84	22
Men (*n* 20)	9·3	7·9, 10·7	0·12	0·16	−0·50, 0·82	−3·2***	3·4	76	24
Both women + men (*n* 40)	8·3	7·5, 9·1	0·40*	0·37	0·09, 0·66	−2·5***	2·9	80	23
MiniMeal-Q									
Women (*n* 20)	7·4	6·2, 8·6	−0·05	−0·07	−0·74, 0·60	−1·6*	3·3	86	35
Men (*n* 20)	9·5	7·8, 11·2	0·17	0·29	−0·52, 1·10	−3·0**	3·9	77	30
Both women + men (*n* 40)	8·5	7·4, 9·5	0·28	0·34	−0·04, 0·72	−2·3***	3·6	82	33
TEE_DLW_									
Women (*n* 20)	9·0	8·1, 9·9	–	–	–	–	–	–	–
Men (*n* 20)	12·5	11·5, 13·6	–	–	–	–	–	–	–
Both women + men (*n* 40)	10·8	9·9, 11·6	–	–	–	–	–	–	–

* *P* < 0·05, ** *P* < 0·01, *** *P* < 0·001.

† Pearson correlation coefficient between measured TEE and reported EI.

‡ The calibration coefficient with 95 % CI corresponds to the slope of the regression of the measured TEE and estimated EI.

**Fig. 2.**
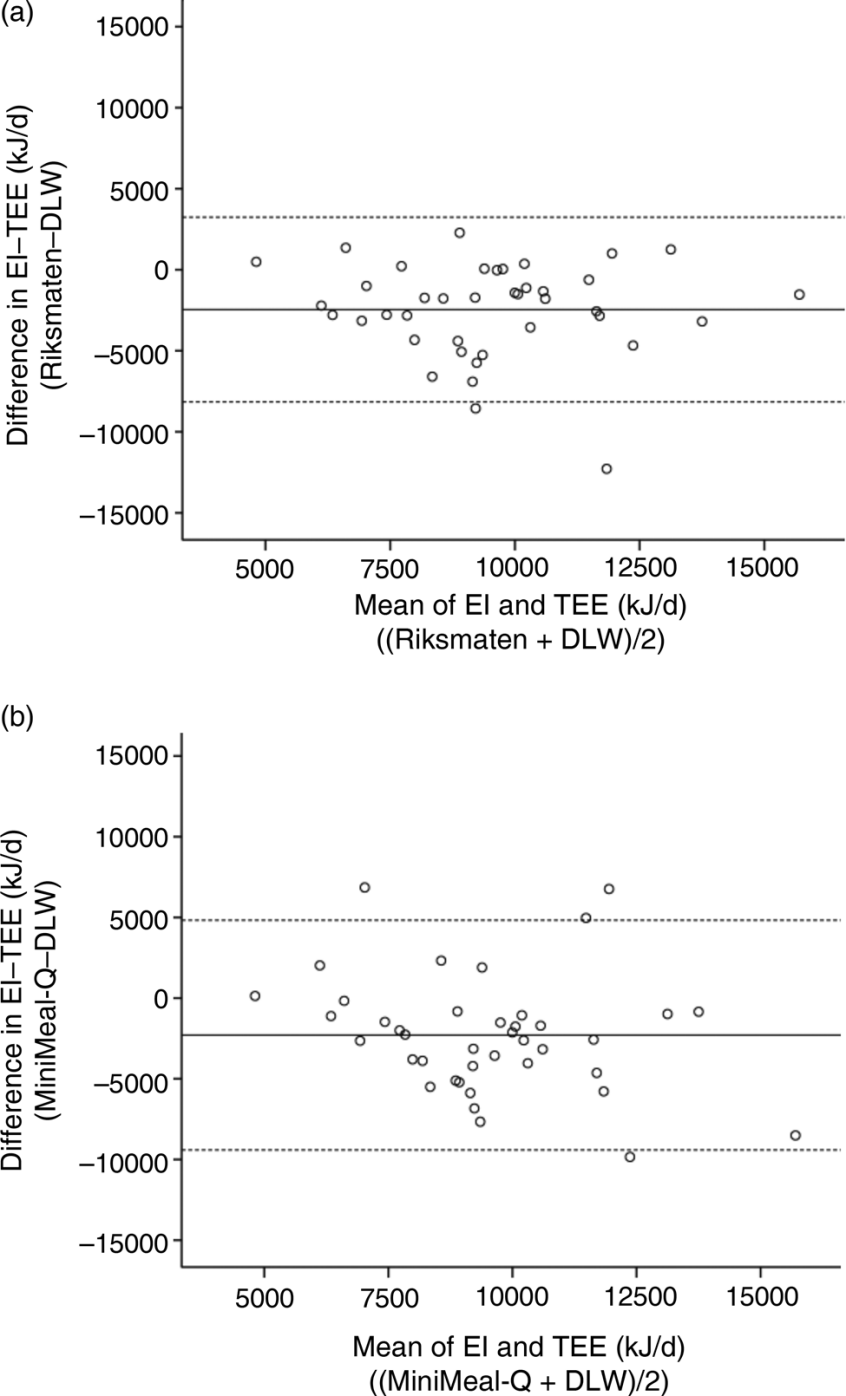
Bland–Altman plots of reported energy intake (EI) from (a) the Riksmaten method and (b) MiniMeal-Q, and total energy expenditure (TEE) measured by doubly labelled water (DLW) in the subgroup (*n* 40). Plots are presented with mean difference of the two methods together with 95 % limits of agreement (mean difference ±1·96 sd of the difference between the methods).

Bland–Altman plots with TEE_DLW_ showed that both the Riksmaten method and MiniMeal-Q underestimated EI (Fig. 2). The limits of agreement were wider for MiniMeal-Q than for the Riksmaten method. The accuracy of reported energy seemed to be similar across the EI ranges for both methods (*P* for trend ns).

The ability to rank individuals according to EI values *v*. TEE_DLW_ was also evaluated with cross-classification analyses. The proportion of participants categorised in the exact same tertile was 47·5 % for the Riksmaten method and 42·5 % for MiniMeal-Q, and the amount categorised in the extreme opposite tertile was 12·5 % for the Riksmaten method and 17·5 % for MiniMeal-Q. The weighted κ for energy was *κ*_w_ = 0·26 for the Riksmaten method and *κ*_w_ = 0·15 for MiniMeal-Q (Table 3).

**Table 3.** Percentages of subjects classified in the same, adjacent and opposite tertiles of energy intake *v*. total energy expenditure (TEE), and relative comparison of energy-adjusted* nutrient intake (the Riksmaten method and MiniMeal-Q) and weighted κ (*κ*_w_)

Energy and macronutrients	Exact agreement (%)	Adjacent (%)	Opposite (%)	*κ*_w_
Energy (*n* 40): Riksmaten – TEE_DLW_†	47·5	40	12·5	0·26
Energy (*n* 40): MiniMeal-Q – TEE_DLW_†	42·5	40	17·5	0·15
Energy (*n* 200): Riksmaten – MiniMeal-Q‡	32	62·5	5·5	0·21
Protein‡ (*n* 200)	28	67	5	0·17
Fat‡ (*n* 200)	33·5	62·5	4	0·25
Carbohydrate (*n* 200)‡	34	62·5	3·5	0·27

DLW, doubly labelled water.

* Energy adjusted by the residual model.

† Subjects who had undergone measurements of TEE. Results of cross-classification analyses of study population divided into tertiles.

‡ Results of cross-classification analyses between the Riksmaten method and MiniMeal-Q, divided into quartiles.

### Comparison of energy and macronutrients between the methods

**Fig. 3.**
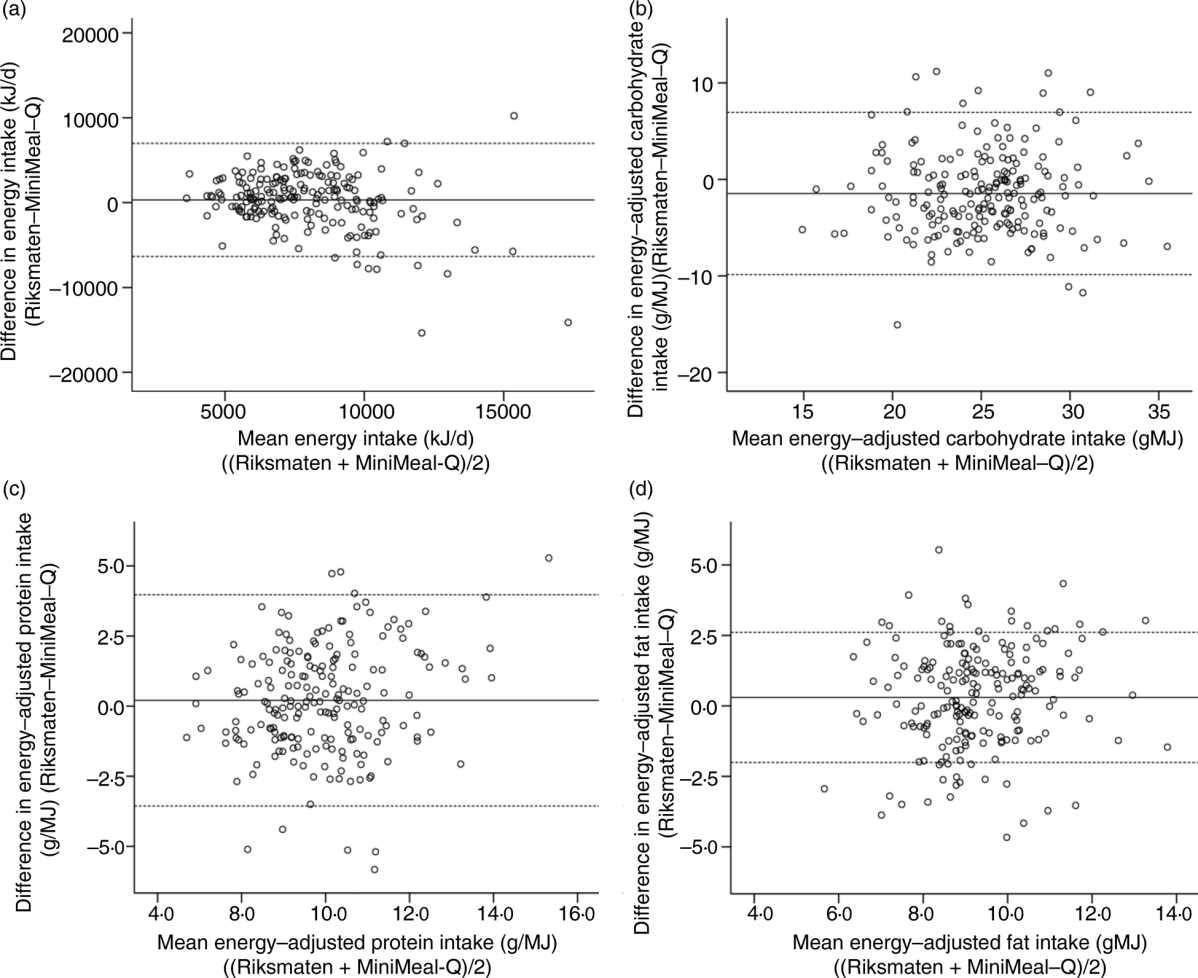
Bland–Altman plots of (a) absolute energy intake and energy-adjusted intake of (b) carbohydrate, (c) protein and (d) fat in all participants (*n* 200) in the Swedish CArdioPulmonary bioImage Study (SCAPIS) diet substudy. Plots are presented with mean difference of the two methods together with 95 % limits of agreement (mean difference ±1·96 sd of the difference between the methods).

Bland–Altman plots of absolute EI and energy-adjusted intakes of carbohydrate, protein and fat between the methods are displayed in Fig. 3(a)–Fig. 3(d). Reported EI was within the limits of agreement for most individuals; however, the differences between the methods seemed to be larger at higher intake levels (Fig. 3(a)). Energy-adjusted carbohydrate (Fig. 3(b)) and protein (Fig. 3(c)) intakes showed acceptable agreement, whereas the agreement for fat intake (Fig. 3(d)) was poorer between the methods (i.e. 18 % captured outside of 95 % limits of agreement).

Reported intakes (mean values and standard deviations and median values with 25th and 75th percentiles) of energy, macronutrients and specific fatty acids, as well as crude and energy-adjusted correlations, are displayed in Tables 4 and 5 for women and men, respectively. In women, energy and macronutrients did not differ significantly between the two methods. However, reported intake of alcohol was significantly higher in the Riksmaten method than in MiniMeal-Q (*P* < 0·001). The average crude correlation was 0·41 (range 0·17–0·71). Pearson energy-adjusted correlations were somewhat higher, 0·44 on average (range 0·20–0·77). For men, reported energy and macronutrient intakes (except for carbohydrate) were significantly higher in the Riksmaten method than in MiniMeal-Q. The average crude correlation was 0·35 (range 0·10–0·67) and increased to 0·43 (range 0·14–0·69) for energy-adjusted values.

**Table 4.** Average daily intake of energy, macronutrients and alcohol for women in the Swedish CArdioPulmonary bioImage Study (SCAPIS) diet substudy (*n* 102) (Riksmaten method and MiniMeal-Q) (Mean values and standard deviations; medians and 25th and 75th percentiles; crude and energy-adjusted (EA) correlations between the methods)

	Riksmaten				MiniMeal-Q						
	Mean	sd	Median	25th and 75th percentiles	Mean	sd	Median	25th and 75th percentiles	*P*†	Crude correlation‡	EA correlation§
Energy (MJ)	7·2	1·9	7·1	5·8, 8·3	7·5	3·3	6·7	5·6, 8·6	0·84	0·33**	
Protein (g)	70	18	71	56, 82	72	30	63	52, 85	0·55	0·39**	0·42**
Fat (g)	69	26	65	54, 82	71	36	64	47, 85	0·65	0·34**	0·31**
SFA (g)	24·8	8·8	23·4	18·6, 29·9	26·2	14·8	22·7	17·1, 32·7	0·93	0·17	0·20*
MUFA (g)	26·5	11·6	24·7	19·3, 32·1	25·7	13·8	23·2	16·2, 31·8	0·18	0·35**	0·40**
PUFA (g)	12·8	7·1	10·9	8·3, 14·6	13·5	8·4	11·6	8·2, 16·7	0·53	0·45**	0·36**
Carbohydrate (g)	176	52	174	139, 214	194	97	176	125, 230	0·27	0·38**	0·47**
Alcohol (g)	9·6	10·5	7·4	0·0, 15·6	5·4	4·8	4·2	1·2, 8·8	<0·001	0·71**	0·77**
Protein (% energy)	16·6	3·2	16·6	14·2, 18·6	16·4	2·7	16·3	14·5, 18·5	0·51	0·39**	
Fat (% energy)	35·8	6·4	35·4	30·9, 40·3	35·3	6·2	25·5	31·7, 38·6	0·48	0·38**	
Carbohydrate (% energy)	41·3	6·9	41·5	36·5, 45·7	43·2	7·2	43·6	38·9, 46·4	0·01	0·45**	
Alcohol (% energy)	3·9	4·1	3·0	0·0, 6·2	2·5	2·6	1·7	0·6, 3·9	<0·001	0·70**	

* *P* < 0·05, ** *P* < 0·01.

† Wilcoxon signed-rank sum test between crude nutrient data.

‡ Spearman correlation coefficient.

§ Pearson correlation coefficient, energy-adjusted by the residual model.

**Table 5.** Average daily intake of energy, macronutrients and alcohol for men in the Swedish CArdioPulmonary bioImage Study (SCAPIS) diet substudy (*n* 98) (Riksmaten method and MiniMeal-Q) (Mean values and standard deviations; medians and 25th and 75th percentiles; crude and energy-adjusted (EA) correlations between the methods)

	Riksmaten				MiniMeal-Q						
	Mean	sd	Median	25th and 75th percentiles	Mean	sd	Median	25th and 75th percentiles	*P*†	Crude correlation‡	EA correlation§
Energy (MJ)	9·1	2·5	9·2	7·4, 10·4	8·2	3·0	7·8	6·1, 10·0	0·002	0·33**	
Protein (g)	91	27	87	76, 102	80	30	78	59, 97	<0·001	0·37**	0·37**
Fat (g)	86	32	66	82, 107	73	30	73	49, 67	0·001	0·28**	0·40**
SFA (g)	31·2	13·0	29·9	22·7, 38·0	27·9	13·9	25·3	18·3, 34·1	0·02	0·34**	0·38**
MUFA (g)	32·9	12·6	30·0	24·7, 40·4	26·4	10·2	26·0	18·6, 31·8	<0·001	0·29**	0·44**
PUFA (g)	15·4	7·5	13·9	10·4, 18·7	12·8	5·9	12·0	8·3, 16·2	0·009	0·10	0·14
Carbohydrate (g)	218	62	223	168, 251	213	88	205	148, 251	0·22	0·33**	0·46**
Alcohol (g)	17·7	18·9	11·6	0·5, 28·6	11·8	9·3	10·3	4·1, 18·8	<0·001	0·67**	0·69**
Protein (% energy)	17·0	3·0	16·8	15·1, 18·4	16·6	2·5	16·0	14·8, 18·2	0·14	0·38**	
Fat (% energy)	35·1	6·2	34·8	31·7, 38·7	33·3	5·0	33·6	30·1, 36·3	0·006	0·39**	
Carbohydrate (% energy)	40·3	7·0	40·5	36·2, 44·6	43·3	6·3	43·8	39·7, 47·1	<0·001	0·47**	
Alcohol (% energy)	5·4	5·5	3·8	0·1, 8·7	4·5	4·0	3·7	1·8, 6·2	0·04	0·64**	

** *P* < 0·01.

† Wilcoxon signed-rank sum test between crude nutrient data.

‡ Spearman correlation coefficient.

§ Pearson correlation coefficient, energy-adjusted by the residual model.

The cross-classification analysis between the methods showed a weighted κ on energy *κ*_w_ = 0·21. For macronutrients, the average *κ*_w_ was 0·23 (range 0·17–0·27). On average 32 % of the participants were categorised in the exact same quartile, while 4·5 % were categorised in the extreme opposite quartile (Table 3).

## Discussion

In this study we examined the ability of two web-based dietary assessment methods, the Riksmaten method and MiniMeal-Q, to assess EI in a middle-aged, population-based sample in relation to DLW. Also, a relative comparison of energy and macronutrients of the Riksmaten method and MiniMeal-Q was performed. The results showed that the mean reporting accuracy was similar for both methods. However, at individual level the correlation and cross-classification analyses suggested that MiniMeal-Q was less successful in ranking individuals correctly on EI, compared with the Riksmaten method.

One of the major strengths with this study is that reported EI has been validated against objectively measured energy expenditure using the ‘gold standard’ DLW. The subgroup that underwent DLW measurements did not differ from the study population in respect to any personal characteristics or by reported EI. We did see a trend that the validity was higher for men in high-SES areas for EI (but unchanged for women) and for BMI, where the reporting accuracy seemed to be better for those with a BMI <30 kg/m^2^. Unfortunately, the DLW method is expensive and only a subgroup of participants could be included in this part of the study. A larger sample size would have been desirable to be able to draw any conclusions from further subgroup analyses. A larger sample size would also have enabled us to calculate validity coefficients and CIs, as at least 100 observations would be preferred for these calculations^(^^27^^,^^28^^)^.

Another strength of this study is the large proportion of participants living in low-SES areas, which makes the study population more heterogeneous in respect to educational level, ethnicity and BMI, for instance. The characteristics of the study population were overall similar to the general population in Sweden^(^^29^^)^, suggesting that the results of this study could be generalised to a middle-aged Swedish population. This is in contrast to many DLW validation studies that use self-selected study participants with a relatively high educational level limiting the generalisability of the results to a broader population^(^^30^^)^. Further, we see no reason to believe that our results would not also be valid for adult populations in other countries with similar socio-economic characteristics.

One limitation of this study is that we were unable to complete replicate measurements of the dietary assessments and the DLW measurements. Replicate measures would provide better data on habitual food intake when using the Riksmaten method, as we know that only 4 d of recording is insufficient for capturing intake of most nutrients^(^^31^^,^^32^^)^. In as much as MiniMeal-Q is a retrospective method, it would have been preferable to repeat the questionnaire after the DLW measurement to be able to reflect the same time period. Still, we do not believe that the habitual diet would change dramatically during those 2 weeks.

Most of the participants managed to report their food intakes by the web, which demonstrate that using web-based dietary assessment methods is feasible in this age group. It is possible that prior computer knowledge affected the willingness to participate in the study, knowing that it included online tasks. Still, the study population was similar to the background population, indicating little effect of self-selection bias.

The measured energy expenditure with DLW was well in line with the age-specific means concluded by Black & Cole^(^^24^^)^. The mean reported EI was around 80 % of measured energy expenditure in both methods, which is coherent with results from traditional paper-based diet records^(^^33^^,^^34^^)^, FFQ^(^^35^^)^, a web-based record tool^(^^36^^)^ and conclusions from reviews^(^^30^^,^^37^^)^. The fact that participants needed to register their dietary intake through the web did apparently not influence the degree of misreporting in either direction. While the average EI precision was similar for both methods, the ranking capacity on the individual level differed; only the Riksmaten method displayed statistically significant correlation on EI *v*. TEE_DLW_. Similar differences were seen for the weighted κ analyses that suggested a fair agreement for the Riksmaten method and a slight agreement for MiniMeal-Q on reported EI *v*. TEE_DLW_. Few validation studies report correlation coefficients and κ statistics, making comparisons difficult. High *r* (0·46–0·77) between reported EI and TEE_DLW_ have been reported in small, highly selective study groups using food records^(^^38^^,^^39^^)^ but also low *r* (0·13) have been reported^(^^40^^)^. However, our results are in line with a recent review that pooled data and showed an average correlation of 0·21 between true intake determined by DLW and estimated EI by FFQ^(^^30^^)^. For MiniMeal-Q the *r* was somewhat lower in this study than in the original validation study (*r* 0·28 *v. r* 0·38)^(^^13^^)^. The original validation study was performed on truncated data in a young, lean and highly educated group, which may explain the somewhat lower correlation in our study compared with the previous study. Reporting bias seems to be predicted by several variables, e.g. age, BMI and level of education, and these differences in characteristics between the two study populations are probably accountable for the different results.

The confidence limits of the Bland–Altman plots were larger for MiniMeal-Q compared with the Riksmaten method, suggesting a lower precision for MiniMeal-Q for assessing absolute EI at the individual level. The lower precision for the FFQ corresponds to the conclusions that were made in the Observing Protein and Energy Nutrition (OPEN) study^(^^41^^)^ and in a study comparing different dietary assessment methodologies using the Portable Electronic Tape Recorded Automatic (PETRA) scales^(^^42^^)^, where it was suggested that FFQ were not suitable for assessing absolute nutrient intakes as they would attenuate true diet–disease risk, but rather suggesting using energy-adjusted values. When adjusting for EI, correlation coefficients for macronutrients improved slightly for both women and men, suggesting that energy-adjusted variables are preferable.

In the present study we did not regard either of the two subjective dietary assessment methods as a reference method. Therefore, we performed relative comparisons between the two methods but did not calculate attenuation factors^(^^43^^,^^44^^)^. Attenuation factors for MiniMeal-Q are, however, presented in the original validation study^(^^13^^,^^14^^)^. The relative comparison between the two subjective methods displayed similar intakes of energy and macronutrients at group level for women, although the variances were larger for MiniMeal-Q. In men, protein and fat intake was higher (and thus the EI) in the Riksmaten method than in MiniMeal-Q. For the agreement between the methods, the Bland–Altman plots illustrated a larger discrepancy when reported EI was higher (and hence, greater convergence on lower EI). This is to be expected, because some individuals consistently report low intakes regardless of method. When using energy-adjusted data for macronutrients, most subjects were predominantly within the 95 % limit of agreement. The cross-classification analysis places approximately on average a third in the exact same quartile and 4·5 % in the opposite quartile. These results correspond to other validation studies of FFQ, where cross-classification analyses against a reference method grossly misplaced on average 4–5·3 % of all macronutrients in opposite quartiles or quintiles^(^^45^^,^^46^^)^.

In summary, both subjective methods validated in this study appear to function reasonably well regarding the mean reporting accuracy. The use of new technology to facilitate the collection of dietary data in large-scale studies seems to work well for middle-aged individuals, regardless of socio-economic status. Still, the degree of misreporting on EI is equal to that of traditional paper-based methods. The ranking capacity appeared to be better in the Riksmaten method, but then again MiniMeal-Q offers a relatively easy method for large data collection with low burden on study subjects. While the time burden is greater with the Riksmaten method, the advantage is that more detailed information is obtained about study participants’ food intake and the distribution of the meals during the day. More detailed data enable researchers to study more specific questions in relation to risk calculation^(^^47^^)^. When choosing the appropriate dietary assessment method for a survey, many aspects need to be taken into consideration: the precision of the intake data needed at the individual- or group level, the time burden of the study participant as well as the cost of managing the survey.

### Conclusions

This study shows that web-based dietary assessment methods could be used for studying dietary intake in women and men of middle age. Nevertheless, the level of data required (i.e. at the individual- or group level) for the study purpose must carefully be considered. According to EI data validated with the DLW technique, both methods displayed similar precision on EI and, consequently, displayed a similar degree of under-reporting. However, MiniMeal-Q was less successful in ranking individuals compared with the Riksmaten method, suggesting that the FFQ should be used with caution if the aim is to assess EI at the individual level. This limitation may also influence ranking on macronutrients. Under-reporting of EI is a major concern in nutrition research with self-reported data, regardless of method. The development of dietary assessment methods to achieve a limited degree of under-reporting is therefore a major challenge for future research.
